# Intentional Stent Fracture to Accommodate Growth After Coarctation Stenting in an 860‐g Neonate

**DOI:** 10.1002/ccd.70291

**Published:** 2025-10-29

**Authors:** Magdalena Cuman, Gernot Buheitel, Peter Ewert, Stanimir Georgiev

**Affiliations:** ^1^ Department of Pediatric Cardiology and Congenital Heart Disease German Heart Center Munich, Lazarettstraße Munich Germany; ^2^ Department of Pediatric Cardiology and Congenital Heart Disease University Hospital Augsburg, Stenglinstraße Augsburg Germany

**Keywords:** napkin‐ring configuration, neonatal aortic coarctation, stent cracking/intentional stent fracture

## Abstract

**Background:**

Congenital aortic coarctation in neonates and children can be managed with transcatheter stent implantation, but this approach raises the issue of how to accommodate the fixed stent structure within the growing vessel.

**Case Summary:**

A 15‐month‐old boy, born prematurely with critical aortic coarctation, was treated at 1 week of age with the implantation of a coronary stent. Subsequently, multiple stent dilatations were needed to adapt it to the growing aortic vessel. At 3 months the patient underwent balloon dilation with the simultaneous implantation of a second stent, and after 1 year an ultra‐high‐pressure balloon dilatation with intentional fracture of the coronary stent was performed.

**Discussion:**

To overcome the problem of stenting a growing vessel, intentional stent fracture (ISF) with ultrahigh‐pressure (UHP) balloon has been introduced and applied in a limited number of patients. In piglet models, the ISF technique was associated with a significant incidence of complications, which were preventable (with the exception of stent fragment embolization) by performing pre‐stenting before ISF.

## 1 Introduction

Stent implantation is now considered the first‐line therapy for aortic coarctation (CoA) in adults [1, 2, 3]. The advantages of this method have led to its use in adolescents and children [4] and, in some centers, even in small infants [5]. One of the limitations of stent use in growing patients is that they require periodic redilation to accommodate the child's somatic growth. Transcatheter intentional stent fracture (ISF) along the longitudinal axis of an undersized stent using ultra‐high‐pressure (UHP) balloons could potentially obviate the need for challenging surgical stent manipulations [6, 7]. Bench testing and in vivo animal studies have shown that overdilation of a stent alters the shape of its individual cells, changing them from a roughly hexagonal shape to a slit‐like rectangular shape, with the circumferential stent struts aligning to form parallel “rings” and potentially creating a napkin‐ring that puts the balloon at risk of rupture and may eventually require surgical intervention as the only means to remove the metallic ring. To prevent this and other complications (i.e. stent collapse, hemodynamic compromise and embolization of stent fragments) the implantation of a second stent into the previously stented area before ISF has been successfully demonstrated in a piglet model [8]. Our group also performed extensive bench‐side testing and a small initial series of children using a standardized ISF approach was recently published [9].

## 2 Case Report

**Figure 1:**
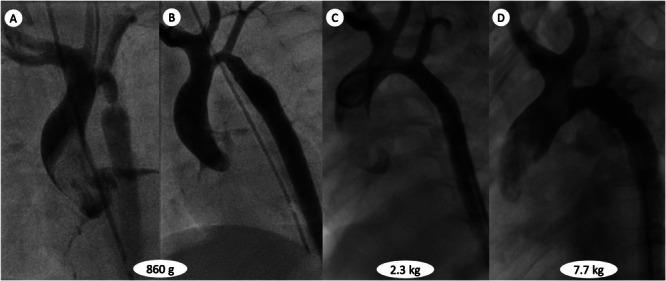
Angiography images of the child's aortic arch configuration over time. Critical aortic coarctation before (A) and after (B) coronary stent implantation. (C) Final result after implantation of the second stent. (D) Anatomy after the intentional cracking of the coronary stent. The hypoplastic transverse arch in a “truncus bicaroticus” anatomy underwent significant growth (transverse to descending aorta ratio increased from 50% up to 100%).

We present the case of a premature twin boy, born at 28 + 1 weeks of gestation through an elective cesarean section in a tertiary perinatal center. At 10 days of life, a significant arm‐to‐leg differential pressure was documented, and echocardiographic examination revealed severe aortic isthmus stenosis. Prostaglandin infusion was promptly started for 72 h and then suspended, since the child was clinically stable and the duct was permanently closed. After 5 days, the child decompensated and required brief resuscitation; prostaglandin was restarted, the patient was intubated, and transferred to our center. At the time of arrival, the child was 2 weeks old with a body weight of 860 g. Clinically, he appeared dystrophic with a reduced general condition. Saturation was good during invasive ventilation; arterial pulses were detectable only in the upper extremities, and an upper‐lower extremities pressure gradient of 35 mmHg was documented. A complete echocardiography evaluation confirmed a hypoplastic aortic arch with a minimal diameter of 1 mm at the isthmus (*z* score −8.5), severe stenosis with a velocity/time index of 3.9 m/s and diastolic runoff. The left ventricle was severely hypertrophic with preserved systolic function. The case was presented and discussed with the cardio‐surgical team, which decided on a percutaneous treatment approach. Because of the high risk of femoral artery closure, the procedure was performed via echo‐guided left carotid artery puncture and insertion of a Leader catheter as first step, then exchanged with a 4 French sheath over a 0.018 Terumo guidewire. Aortic angiography showed an anatomical truncus bicaroticus arch with hypoplastic transverse arch and severe isthmus stenosis. A modified Amplatz Right Catheter with a 0.014 coronary wire was advanced into the descending aorta, and a coronary stent (Onyx 3.5 × 8 mm) was implanted at 18 atm (corresponding to 3.8 mm stent diameter) into the stenotic segment with a good final result. Cardiovascular function was mantained with low‐dose inotropic drug infusion (milrinone and noradrenalin) and low‐dose anticoagulation therapy. Echocardiography confirmed stable stent positioning and a maximal velocity/time index value of 3 m/s. After 48 h, the patient was transferred back to the referring clinic. After 3 months, the patient was electively admitted to our center for enlargement of the aortic stent. At that time, the patient was clinically stable, with a body weight of 2.3 kg, and echocardiography showing accelerated flow at the stent site (velocity/time index 3.8 m/s) with diastolic run‐off. The catheterization procedure was performed under sedation. Though a 5 French Slender sheath in the right femoral artery, we measured a peak‐to‐peak gradient of 30 mmHg at the isthmus; the left ventricular end diastolic pressure (LVEDP) was 16 mmHg. Angiographically, the transverse arch was still hypoplastic (4 mm, *z* score −4.17), the minimal diameter proximal to the stent was 2 mm, and the abdominal aorta measured 5 mm. Over a 0.018” wire, a 5 × 12 mm Formula stent was implanted within the coronary stent and postdilated with a 5 mm Emerge Balloon. The left subclavian artery origin was partially covered by the stent struts without flow interference or residual gradient. The child was discharged 2 days later in good clinical condition, with echocardiography showing minimal antegrade acceleration at the isthmus stents (2.5 m/s) under antiplatelet therapy. At the age of 15 months and a body weight of 7.7 kg, a third percutaneous procedure was electively performed. Through a 5 French Slender sheath, pressures were measured as first step. The gradient through the aortic stent was 16 mmHg, and the LVEDP was 23 mmHg. Aortic angiography showed a minimal in‐stent diameter of 4 mm, post‐stenotic aortic dilatation of 8 mm, ascending aorta measuring 11.5 mm, transverse arch 6 mm and descending aorta 6 mm. We decided to perform a controlled and progressive dilatation of the stents to enlarge the Formula stent to the appropiate aortic diameter and consequently intentionally fracuture the underling coronary stent. A Powerflex 6 mm balloon dilated to 15 atm as the first approach; afterwards, an 8 mm Conquest balloon was dilated up to 30 atm. At this point, the coronary stent was clearly at risk of forming the feared napkin‐ring configuration, and the Formula stent shortened from 12 to 8 mm. After performing angiography that excluded any vessel injury, the balloon was inflated up to 40 atm achieving complete cracking of the coronary stent at about 35 atm when the balloon eliminated the waist produced by the stent ring (Supporting Information S1: Video). The angiographic and hemodynamic final result were optimal, with no pressure gradient at the stent site and a harmonious anatomical configuration of the aortic arch (Figure 1). The patient was discharged the following day with the recommendation for regular follow‐up evaluations and renewed catheterization if a significant gradient develops.

## 3 Discussion

Placement of stents in neonates and small infants inevitably requires multiple transcatheter reinterventions to accommodate ongoing somatic growth. In this context, access‐related vascular injury remains a relevant concern, with a recent study reporting an incidence of 14% in patients younger than 3 months [10]. As early as the 1990s, Mullins stated that stents should only be placed in children if they can be dilated to an adult‐sized diameter appropriate for the target vessel [11]. Over the past three decades, however, stent technology has advanced considerably. Numerous additional stents have become available, most of which are now premounted onto balloon angioplasty catheters. These premounted stents generally exhibit lower profiles and can be delivered through small sheaths. Nevertheless, the majority of them cannot be expanded to adult diameters when implanted in central vessels. More recently, the Renata Minima stent (Renata Medical, Newport Beach, CA, USA) has been introduced [12]. This device was specifically designed for implantation at very small vessel diameters (≥5.1 mm), while still allowing redilation to adult size, and thus potentially offers an alternative to conventional coronary or Formula stents. Although immediate and short‐term results are encouraging, longer follow‐up and a larger study group are still required to confirm its long‐term efficacy and safety. ISF has been clinically reported since the early 2000s, with the first description provided by Maglione et al. in 2009 [6]. Furthermore, several in vitro studies have demonstrated that small‐ and medium‐sized stents can be intentionally fractured using UHP balloons [13]. Building on this evidence, Agrawal et al. reported a series of 22 patients who underwent attempted ISF in various arterial and venous locations, with a low risk of complications. This approach therefore avoided the need for complex surgical stent manipulations [14]. In addition, bench‐testing data [15, 16, 17] showed that balloon re‐dilation technique can significantly influence both stent shortening and the chances of fracture, representing an important factor to consider when planning ISF. Furthermore, the subsequent piglet model developed by Bratincsák et al. [8] provided additional mechanistic insights and experimental validation. Re‐stenting at the time of stent fracture was described clinically by Morray et al. [7]. More recently, our group reported the use of simultaneous pre‐stenting in the context of ISF [9]. In the present case, redilation of the Onyx stent alone was initially considered but ultimately deferred due to concerns regarding potential vessel wall injury and restenosis. Consequently, a pre‐stenting approach using a Formula stent was chosen [9]. Despite stepwise dilatation with progressively large balloons and the deployment of an additional stent, a napkin‐ring‐like narrowing nearly developed. Nevertheless, the stent could be fractured with UHP dilatation, resulting in an excellent clinical and angiographic result and restoring a harmonious anatomical configuration of the entire aortic arch.

## Consent

The authors confirm that written consent for the submission and publication of this case, including images, has been obtained from the patient in line with COPE guidance.

## Conflicts of Interest

The authors declare no conflicts of interest.

## Supporting information


**Video legend:** Intentional fracturing of the coronary stent applying 35 atm using an ultra‐high‐pressure balloon. pre‐ and post‐ISF aortic arch angiography.
